# Association between dietary glycine intake and the prevalence of hypertension, hyperlipidemia, overweight or obesity in rural northern China: a cross-sectional study

**DOI:** 10.3389/fnut.2024.1364309

**Published:** 2024-07-12

**Authors:** Ying Feng, Xing-bo Gu, Meng Zhou, Hong-lan Wang, Ren-nan Feng, Zhi-hong Zhang

**Affiliations:** ^1^Department of Nutrition and Food Hygiene, School of Public Health, Heinz Mehlhorn Academician Workstation, Hainan Medical University, Haikou, Hainan, China; ^2^Department of Nutrition and Food Hygiene, Harbin Medical University, Harbin, China

**Keywords:** dietary glycine, overweight, obesity, hypertension, hyperlipidemia, oxidative stress

## Abstract

**Objective:**

The objective of this research is to investigate the relationship between dietary glycine consumption and the prevalence of hypertension, hyperlipidemia, and overweight or obesity in economically disadvantaged areas of northern China using a cross-sectional study design.

**Methods:**

A cross-sectional study involving 774 participants utilized a web-based dietary questionnaire (IDQC) and underwent physical measurements. Data analysis was conducted using IBM SPSS Statistics software (Version 21). Participants were stratified into four groups based on quartiles of their dietary glycine intake: Q1 (<1.32), Q2 (1.32–1.82), Q3 (1.82–2.26), and Q4 (>2.26). Continuous variables were reported as mean ± standard deviation and compared using ANOVA or the Kruskal-Wallis test, while categorical variables were presented as frequencies (%) and compared using the chi-square test. Finally, multivariable logistic regression with *p*-value of less than 0.05 was considered statistically significant.

**Results:**

Significant differences in dietary glycine intake were observed between the highest quartile group (Q4) and the lowest quartile group (Q1), with corresponding dominance ratios of 0.590 (95% CI, 0.360–0.966), 0.547 (95% CI, 0.327–0.913), and 0.547 (95% CI, 0.353–0.850) for the risk of hypertension, hyperlipidemia, and overweight/obesity, respectively. Furthermore, no significant correlation was found between dietary glycine intake and hypertension or hyperlipidemia within each sex and age subgroup.

**Conclusion:**

There exists a potential correlation between increased dietary glycine intake and reduced prevalence of hypertension, hyperlipidemia, and overweight/obesity. However, additional research is necessary to validate this finding through larger-scale studies conducted at a population level.

## 1 Introduction

Given the evolving disease landscape and lifestyle modifications, hypertension, overweight/obesity, and dyslipidemia have emerged as significant risk factors for numerous prevalent chronic ailments such as hypertension, cancer, and diabetes. The prevalence of these conditions has been steadily rising in recent years, posing a substantial global public health concern (1–7). Studies have shown an association between dietary factors and the occurrence and development of chronic diseases (8, 9). Currently, there are more studies on salt intake, vegetable and fruit intake, and cereal intake (10–19), but fewer studies on dietary intake of amino acids, especially glycine.

Glycine is the lowest-molecular-weight amino acid in proteins. The human body can synthesize glycine endogenously, but in some cases, glycine synthesis *in vivo* may not be sufficient to meet the metabolic needs of body tissues (20, 21). Several studies have found that in addition to its involvement in the composition of proteins, glycine is also involved in the regulation of gene expression (22, 23) and the synthesis of glutathione (24). Chronic glycine deficiency may impair the health status of the organism. Glycine may be involved in reducing the production of free radicals, protecting the organism against damage caused by oxidative stress (25, 26), increasing the production of nitric oxide (27), and synthesizing collagen and elastin (28), among others, thus reducing the incidence of hypertension; Glycine is now recognized as a serum marker for metabolic abnormalities associated with obesity (29), and plasma levels of glycine are found to be lower in obese patients than in healthy individuals (30). Previous animal studies have confirmed that in male mice, glycine intervention improves lipid levels in experimental rats, thereby reducing hypertension and central obesity (31–33); glycine has a protective effect on the elevation of circulating non-esterified fatty acids induced by sucrose intake in male rats (34); and glycine supplementation given to hypercholesterolemic rats results in a significant increase in total nitric oxide (NO) concentration and a certain degree of reduction in blood cholesterol levels and triglyceride levels (35).

Although there are associations between glycine and a variety of chronic diseases, most of the available studies have been limited to animal models or plasma levels of glycine and related chronic diseases, the relationship between glycine consumption and concomitant chronic diseases is less well-known. In other words, the relationship between dietary intake of glycine and the prevalence of hypertension, hyperlipidemia, and obesity remains unclear. In turn, it is not possible to determine the causal relationship between glycine and these chronic diseases and their associated pathological changes. Therefore, the relationship between dietary glycine intake and chronic diseases is worth investigating.

This study investigates the relationship between dietary glycine intake and the prevalence of hypertension, hyperlipidemia, and overweight/obesity by conducting dietary surveys, chronic disease surveys, physical measurements, and collection of blood samples, including the collection of demographic information, lifestyles, dietary habits, and physical indicators of the respondents from the poverty-stricken areas in northern China, to fill in the gaps of the research in this direction and to provide practical nutritional education and dietary guidance for the residents of the poverty-stricken areas.

## 2 Materials and methods

### 2.1 Design of the questionnaire

This study is a population-based cross-sectional study. A simple and user-friendly web-based dietary questionnaire was designed to accurately investigate the dietary habits of the survey respondents. In this questionnaire, common foods were categorized into 16 categories. To accurately assess food intake, all food items were accompanied by pictures of a certain weight or volume for reference. Respondents were asked to indicate the frequency and quantity of each food subcategory consumed across all food groups. Each participant was able to obtain feedback on his/her dietary intake after completing the questionnaire. The studies involving human participants were reviewed and approved by the Harbin Medical University Ethical Review Board. The patients/participants provided their written informed consent to participate in this study.

### 2.2 Reliability and validity of the questionnaire

Several previous research studies have confirmed the reliability and validity of this type of questionnaire. By comparing the survey data of this questionnaire with the results of the three-day dietary record, it has been confirmed that both findings are in good agreement. That is, the questionnaire can be used in large-scale population surveys.

### 2.3 Selection and exclusion criteria for survey respondents

Non-pregnant women aged ≥18 years were randomly selected as survey respondents in Baiquan County, Heilongjiang Province, from 4 to 6 March 2020. Two thousand and five hundred respondents were recruited, of whom 1,170 agreed to register for an account, fill out the questionnaire and cooperate in the collection of physical measurements and blood samples. All survey respondents were registered at www.yyjy365.org/diet, and to ensure that there were no duplicate registrations, each participant was given a specific number as a registered account. All the respondents completed the informed consent form on the web. The School of Public Health Medical Centre approved this study, and all survey operations followed relevant guidelines and regulations.

**Figure 1:**
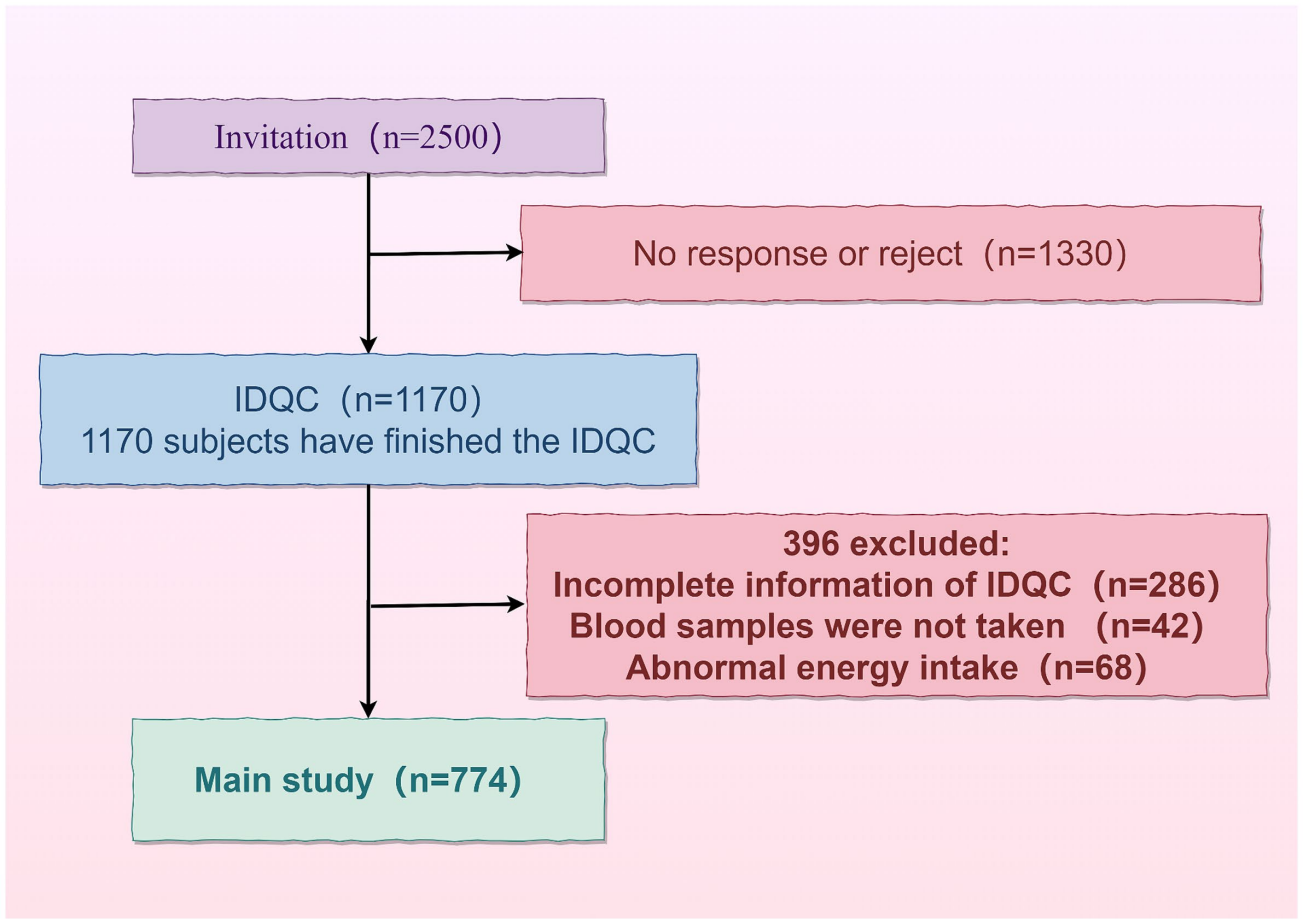
Flowchart for the selection of the population.

The exclusion criteria:

1. incomplete information in the questionnaire;
2. no blood sample taken;
3. non-standardized energy intake [males: <800 kcal (3,349 kJ) or >5,000 kcal (20,934 kJ); females: <600 kcal (2,512 kJ) or >4,000 kcal (16,747 kJ)] (Figure 1).

### 2.4 Estimation of dietary nutrient intake

All the subjects included in the study completed the questionnaire based on their diet in the past year. The frequency and quantity of consumption were filled in detail for each food item. The frequency of consumption of each food item was categorized into eight groups: never (<1 time/month), 1–3 times/month, 1 time/week, 2–3 times/week, 4–5 times/week, 1 time/day, 2 times/day, 3 times/day and more. For most of the food items, except for never consumption, the intake corresponding to each consumption frequency was divided into six categories: less than or equal to 1 tael (1 tael = 50 g), 2–3 tael, 4–5 tael, 6–7 tael, 8–9 tael, and 1 catty and above. In turn, calculate the average daily intake of various nutrients (including the daily dietary intake of glycine) based on the nutrient content of each food in the Chinese Food Composition Table.

### 2.5 Anthropometric measurements and laboratory tests

Measurements of height, weight, systolic blood pressure, diastolic blood pressure, and other indicators were taken by uniformly trained investigators according to standards. Subjects were asked to dress lightly and remove their shoes for height measurements.


$$\mathrm{Body}\;\mathrm{mass}\;\mathrm{index}\;\left(BMI\right)=\;\frac{\mathrm{weight}\;\left(\mathrm{kg}\right)}{\mathrm{height}\;\left(m^{2}\right)}$$

Subjects’ blood pressure was measured twice by a qualified internist, with an interval of at least 30 s between these two independent measurements, and then the average of the blood pressure obtained from the two measurements was taken as the finalized blood pressure value and recorded.

All the study subjects started fasting after 10:00 p.m. on the day before the survey and completed the collection of blood samples by 9:00 a.m. on the day of the survey. The main indexes of the blood samples were total cholesterol (TC), triglyceride (TG), and so on.

### 2.6 Anthropometric measurements and Laboratory tests

1. Overweight: 24 ≤ BMI ≤ 27.9 kg/m^2^; Obesity: BMI ≥ 28 kg/m^2^ (36).
2. Hypertension: systolic blood pressure ≥ 140 mmHg and/or diastolic blood pressure ≥ 90 mmHg or taking antihypertensive drugs (37).
3. Hyperlipidemia: blood total cholesterol ≥6.2 mmol/L and/or blood triglycerides ≥1.7 mmol/L or taking lipid-lowering drugs (38).

### 2.7 Statistical analysis

The statistical analysis was conducted utilizing IBM SPSS Statistics software (Version 21). The participants were categorized into four groups based on quartiles of their dietary glycine intake: Q1 (<1.32), Q2 (1.32–1.82), Q3 (1.82–2.26), and Q4 (>2.26). Continuous variables were presented as the mean ± standard deviation and were compared using either ANOVA or the Kruskal-Wallis test. Categorical variables were expressed as frequencies (%) and were compared using the chi-square test.

To elucidate the distinct correlations between dietary glycine intake and hypertension, overweight/obesity, and hyperlipidemia, three multivariate logistic regression models were constructed. The first model (utilizing Q1 as the baseline group) was adjusted for age, gender, labor intensity, income, literacy, and energy intake. The second model incorporated smoking and alcohol consumption as additional variables to model 1. Lastly, the third model included the total dietary salt intake in model 2. Furthermore, we conducted stratified analyses based on age (<60 and ≥60 years) and gender subgroups, while also accounting for confounding variables in the multivariate analyses (excluding stratification). This approach aimed to evaluate potential variations in the relationship between dietary glycine intake and the occurrence of hypertension, overweight/obesity, and hyperlipidemia. Statistical significance was determined by considering two-sided *р* values < 0.05 in all analyses.

## 3 Results

The prevalence of hypertension was 29.8%, overweight/obesity 44.4%, obesity 8.9%, and hyperlipidemia 35.3% among the 774 survey respondents. The results of group comparisons at the quartile level of dietary glycine intake showed that the differences between the four groups in labor intensity, smoking, BMI, alcohol consumption, monthly income, education level, systolic blood pressure, total dietary energy intake, total dietary protein intake, total fat intake, total carbohydrate intake, dietary fiber intake, cholesterol intake, fatty acid intake, and saturated fatty acid intake were statistically significant (all *p* < 0.05), as shown in Table 1.

**Table 1:** Characteristics of participants according to quartiles of dietary glycine intake.

	Quartiles of glycine				*p*
Glycine, g (energy-adjusted)	Q1:<1.32	Q2:1.32–1.82	Q3:1.82–2.26	Q4:>2.26	
Participants, *n*	193	194	194	193	
Age, year	60.1 ± 11.1	62.4 ± 11.5	61.1 ± 11.8	60.0 ± 11.1	0.133
**Gender**					0.635
Male, *n* (%)	110 (57.0)	111 (57.2)	103 (53.1)	115 (59.6)	
Female, *n* (%)	83 (43.0)	83 (42.8)	91 (46.9)	78 (40.4)	
**Labor-intensity**					0.004
Light, *n* (%)	32 (16.6)	66 (34.0)	45 (23.2)	39 (20.2)	
Medium, *n* (%)	10 (5.2)	11 (5.7)	13 (6.7)	13 (6.7)	
Heavy, *n* (%)	151 (78.2)	117 (60.3)	136 (70.1)	141 (73.1)	
**Smoking**					0.046
Never smoking, *n* (%)	126 (65.3)	139 (71.6)	131 (67.5)	152 (78.8)	
Quit smoking, *n* (%)	11 (5.7)	14 (7.2)	19 (9.8)	7 (3.6)	
<10 zhi/day, *n* (%)	44 (22.8)	30 (15.5)	35 (18.0)	22 (11.4)	
≥10 zhi/day, *n* (%)	12 (6.2)	11 (5.7)	9 (4.6)	12 (6.2)	
**Drinking**					<0.001
Nondrinker, *n* (%)	134 (69.4)	162 (83.5)	171 (88.1)	161 (83.4)	
Current-drinker, *n* (%)	59 (30.6)	32 (16.5)	23 (11.9)	32 (16.6)	
**Income per month**					<0.001
<1,000 yuan, *n* (%)	112 (58.0)	188 (96.9)	173 (89.2)	157 (81.3)	
≥1,000 yuan, *n* (%)	81 (42.0)	6 (3.1)	21 (10.8)	36 (18.7)	
**Education**					<0.001
≤secondary school, *n* (%)	84 (43.5)	138 (71.1)	148 (76.3)	141 (73.1)	
≥junior high school, *n* (%)	109 (56.5)	56 (28.9)	46 (23.7)	52 (26.9)	
SBP (mmHg)	131.1 ± 12.7	130.4 ± 11.8	128.2 ± 11.2	128.3 ± 11.2	0.026
DBP (mmHg)	82.8 ± 8.7	82.3 ± 7.4	81.3 ± 8.2	81.4 ± 7.0	0.167
Hypertension	129 (66.8)	130 (67.0)	136 (70.1)	148 (76.7)	0.119
TG (mmol/l)	1.7 ± 1.3	1.6 ± 1.5	1.5 ± 1.2	1.5 ± 1.6	0.829
TC (mmol/l)	5.0 ± 1.1	5.0 ± 1.0	5.1 ± 1.1	4.9 ± 1.1	0.237
High TG/TC	116 (60.1)	124 (63.9)	127 (65.5)	134 (69.4)	0.286
Weight, kg	136.3 ± 22.4	123.7 ± 18.2	127.1 ± 18.6	128.2 ± 20.8	<0.001
BMI, kg/m^2^	24.6 ± 3.2	23.0 ± 2.9	23.7 ± 3.0	23.6 ± 3.2	<0.001
**BMI categories**					0.001
<24.0, *n* (%)	83 (43.0)	125 (64.4)	106 (54.6)	116 (60.1)	
24.0–27.9, *n* (%)	84 (43.5)	57 (29.4)	72 (37.1)	62 (32.1)	
≥28.0, *n* (%)	26 (13.5)	12 (6.2)	16 (8.2)	15 (7.8)	
**Dietary intakes**					
Energy, kcal/day	2641.4 ± 1062.6	1742.0 ± 741.3	1892.1 ± 517.8	2551.1 ± 797.4	<0.001
Total protein, g/day	67.9 ± 27.2	53.7 ± 22.1	64.7 ± 16.7	97.8 ± 28.1	<0.001
Total fat, g/day	50.0 ± 28.4	45.7 ± 28.8	56.0 ± 19.0	84.3 ± 28.9	<0.001
Total carbohydrate, g/day	482.8 ± 208.7	283.5 ± 131.0	294.6 ± 105.8	368.6 ± 150.8	<0.001
Fiber, g/day	13.2 ± 5.8	11.7 ± 4.9	15.0 ± 5.6	23.9 ± 9.3	<0.001
Cholesterol, mg/day	177.5 ± 137.4	275.2 ± 285.8	310.7 ± 187.8	509.3 ± 347.2	<0.001
Salt, g/day	7.5 ± 6.4	6.5 ± 3.5	6.6 ± 6.5	7.0 ± 5.0	0.233
Fatty acid, g/day	30.7 ± 23.8	27.9 ± 24.8	34.6 ± 13.9	53.8 ± 24.9	<0.001
SFA, g/day	5.3 ± 3.9	5.1 ± 4.0	6.6 ± 2.6	10.3 ± 4.6	<0.001

^*^SBP, systolic blood pressure; DBP, diastolic blood pressure; TG, triglycerides; TC, total cholesterol; BMI, body mass index; SFA, saturated fatty acids.

**Figure 2:**
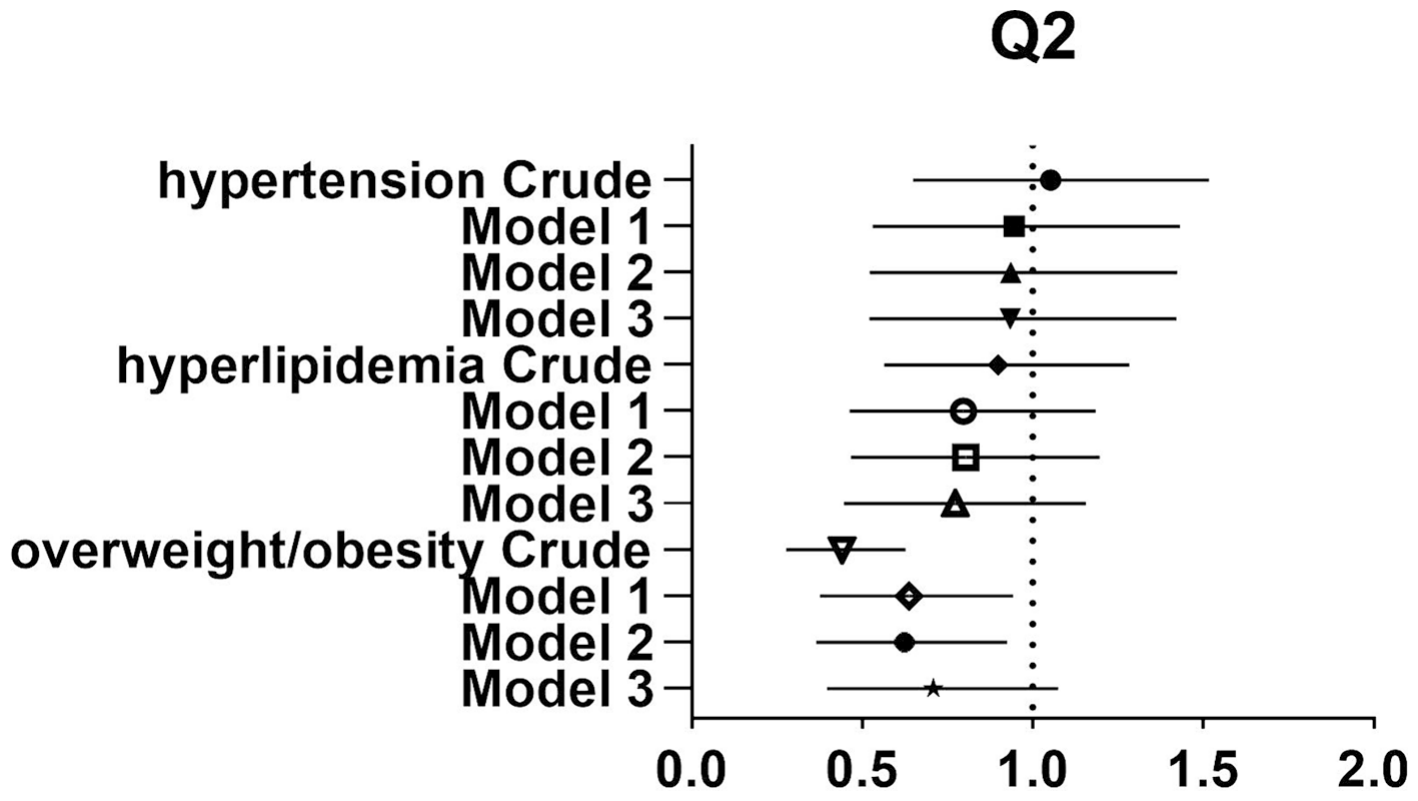
Forest plot of prevalence and 95% CIs for hypertension, hyperlipidaemia and overweight/obesity based on the Q2 of dietary glycine intake.

**Figure 3:**
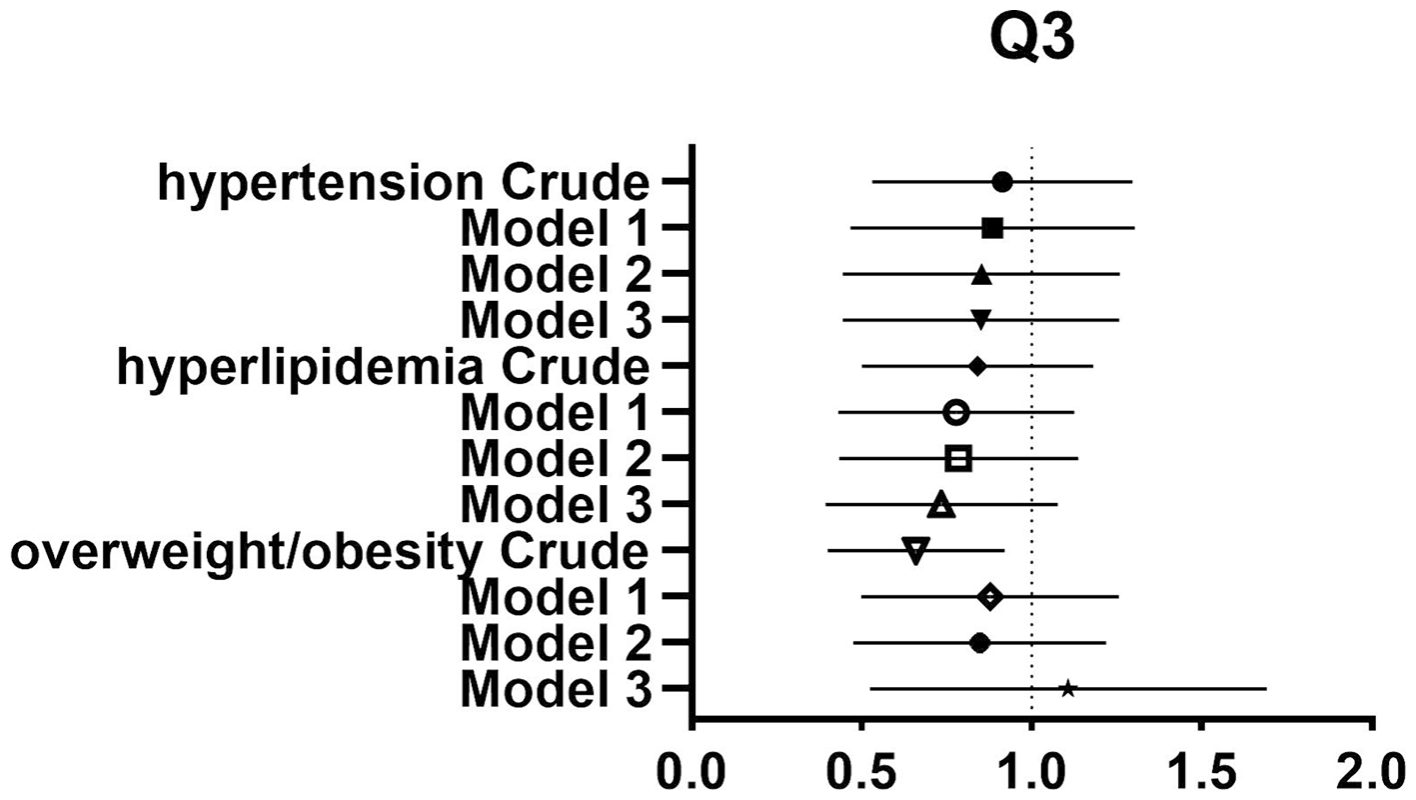
Forest plot of prevalence and 95% CIs for hypertension, hyperlipidaemia and overweight/obesity based on the Q3 of dietary glycine intake.

**Figure 4:**
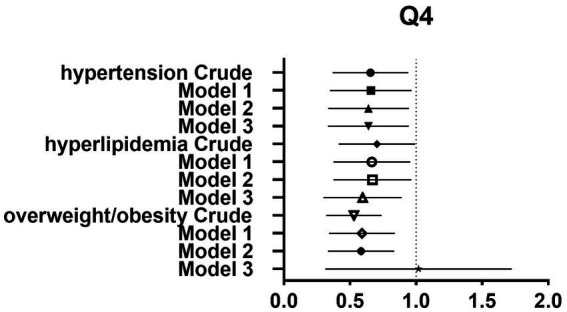
Forest plot of prevalence and 95% CIs for hypertension, hyperlipidaemia and overweight/obesity based on the Q4 of dietary glycine intake.

After controlling for potential confounders such as age, sex, labor intensity, income, literacy, energy intake, smoking, alcohol consumption, and total dietary salt intake, a significant difference in dietary glycine intake was observed between the highest quartile group (Q4) and the lowest quartile group (Q1). The dominance ratios for the risk of hypertension, hyperlipidemia, and overweight/obesity were 0.590 (95% CI, 0.360–0.966; *p* < 0.05), 0.547 (95% CI, 0.327–0.913; *p* < 0.05), and 0.547 (95% CI, 0.353–0.850; *p* < 0.05), respectively. Further details can be found in the accompanying Table 2 and Figures 2–4.

**Table 2:** Odds ratios and 95% CIs of hypertension, hyperlipidemia, and overweight/obesity according to quartiles of dietary glycine intake.

	Dietary glycine intake			
	Q1: <1.32	Q2: 1.32–1.82	Q3: 1.82–2.26	Q4: >2.26
**Hypertension**				
Crude	1	0.992 (0.650, 1.516)	0.860 (0.560, 1.320)	0.613 (0.391, 0.960)^*^
Model 1	1	0.872 (0.531, 1.432)	0.819 (0.504, 1.332)	0.609 (0.375, 0.988)^*^
Model 2	1	0.863 (0.523, 1.423)	0.787 (0.481, 1.288)	0.591 (0.361, 0.967)^*^
Model 3	1	0.861 (0.521, 1.421)	0.786 (0.480, 1.286)	0.590 (0.360, 0.966)^*^
**Hyperlipidemia**				
Crude	1	0.850 (0.564, 1.283)	0.795 (0.526, 1.201)	0.663 (0.436, 1.010)
Model 1	1	0.741 (0.464, 1.185)	0.726 (0.460, 1.148)	0.623 (0.398, 0.974)*
Model 2	1	0.747 (0.466, 1.196)	0.733 (0.463, 1.161)	0.626 (0.399, 0.984)*
Model 3	1	0.718 (0.446, 1.155)	0.682 (0.423, 1.099)	0.547 (0.327, 0.913)*
**Overweight/obesity**				
Crude	1	0.417 (0.277, 0.627)***	0.626 (0.419, 0.936)*	0.501 (0.334, 0.751)**
Model 1	1	0.595 (0.376, 0.942)*	0.823 (0.529, 1.280)	0.555 (0.361, 0.854)**
Model 2	1	0.581 (0.365, 0.925)*	0.793 (0.506, 1.243)	0.547 (0.353, 0.850)**
Model 3	1	0.653 (0.397, 1.074)	1.004 (0.582, 1.735)	0.855 (0.409, 1.791)

^*^ (1) model 1: adjusted for age, gender, labor intensity, income, literacy level, and energy intake; model 2: adjusted for model 1 plus smoking and alcohol consumption; model 3: adjusted for model 2 plus dietary protein, fat, carbohydrate, dietary fiber, and cholesterol intake.

(2) *p < 0.05; **p < 0.01; ***p < 0.001.

Moreover, a multivariable-adjusted stratified analysis indicated that there was no significant association between dietary glycine intake and hypertension or hyperlipidemia within subgroups categorized by age and sex. However, a statistically significant difference in overweight/obesity was observed between the highest quartile (Q4) and lowest quartile (Q1) groups for dietary glycine intake in females and individuals over the age of 60, with dominance ratios of 0.459 (95% CI, 0.229–0.919; *p* < 0.05) and 0.422 (95% CI, 0.234–0.769; *p* < 0.01), respectively, as presented in Table 3.

**Table 3:** The odds ratios and 95% CIs of hypertension, hyperlipidemia, and overweight/obesity in subgroup analyses.

	Dietary glycine intake			
	Q1: <1.32	Q2: 1.32–1.82	Q3: 1.82–2.26	Q4: >2.26
**Hypertension**				
**Gender**				
Men	1	0.877 (0.455, 1.688)	0.861 (0.450, 1.647)	0.681 (0.350, 1.325)
Women	1	1.208 (0.507, 2.875)	0.995 (0.434, 2.280)	0.523 (0.226, 1.214)
**Age**				
<60	1	0.655 (0.262, 1.637)	0.843 (0.352, 2.022)	0.478 (0.199, 1.151)
≥60	1	1.094 (0.593, 2.020)	0.783 (0.429, 1.430)	0.589 (0.319, 1.085)
**Hyperlipidemia**				
**Gender**				
Men	1	0.826 (0.439, 1.552)	0.655 (0.342, 1.253)	0.686 (0.346, 1.363)
Women	1	0.712 (0.328, 1.548)	0.778 (0.366, 1.655)	0.508 (0.224, 1.148)
**Age**				
<60	1	1.221 (0.589, 2.534)	0.943 (0.452, 1.966)	0.620 (0.287, 1.338)
≥60	1	0.849 (0.444, 1.623)	0.653 (0.334, 1.277)	0.548 (0.260, 1.156)
**Overweight/obesity**				
**Gender**				
Men	1	0.748 (0.383, 1.460)	1.047 (0.489, 2.241)	0.753 (0.266, 2.128)
Women	1	0.579 (0.255, 1.317)	1.043 (0.437, 2.486)	0.842 (0.268, 2.647)
**Age**				
<60	1	1.204 (0.524, 2.768)	1.754 (0.694, 4.434)	2.394 (0.648, 8.844)
≥60	1	0.406 (0.040, 4.093)	0.665 (0.064, 6.881)	1.196 (0.773, 1.850)

* (1) adjusted for age, gender, labor intensity, income, literacy level, energy intake, smoking, alcohol consumption, dietary protein, fat, carbohydrate, dietary fiber, and cholesterol intake; (2) *p < 0.05; **p < 0.01; ***p < 0.001.

## 4 Discussion

In this study, the researchers conducted the first web-based questionnaire study among residents of improved areas in northern China to investigate the relationship between dietary glycine intake and the prevalence of common chronic diseases. The findings revealed a lower prevalence of hypertension, overweight/obesity, and hyperlipidemia among participants with higher dietary glycine intake. Additionally, negative correlations were observed between dietary glycine intake and body weight, BMI, systolic blood pressure, diastolic blood pressure, and blood triglyceride levels.

This investigation suggests that the correlation between dietary glycine intake and overweight/obesity may be stronger in people over 60 years of age compared to those under 60 years of age. The various biological functions of glycine in protection from oxidative stress, regulation of inflammatory responses, and collagen, hemoglobin and bilirubin synthesis may directly or indirectly contribute to the mitigation of vascular damage. Among those, lowering oxidative stress and inflammation have been suggested to provide a link between glycine intake and cardiovascular health. Furthermore, in individuals with obesity, the oxidative stress and inflammation linked to this condition can be alleviated by regulating fatty acid metabolism and enhancing levels of the antioxidant glutathione. Utilizing a homeostatic model of insulin resistance, Takashina and colleagues discovered a positive correlation between glycine concentration and insulin sensitivity and a negative correlation with insulin resistance (45). Significantly, among all the plasma amino acids examined individually, glycine exhibited the strongest association with altered insulin sensitivity (46, 47), thereby mitigating obesity through the facilitation of efficient sugar utilization and reduction of blood glucose levels. Glycine has been found to impact the composition of gut flora, thereby potentially influencing energy metabolism and fat storage in the host. Research indicates that glycine significantly alters gut flora composition and is linked to weight loss. Elevated activity of the glycine degradation pathway in oral and intestinal flora may result in reduced serum glycine levels and the promotion of insulin resistance (48). The intricate and diverse mechanisms linking glycine to chronic diseases, particularly obesity, in the elderly warrant further investigation. Your insights have sparked new avenues for exploration in our research, prompting us to delve deeper into these mechanisms. Thank you for sharing your knowledge.

Not only was the association between dietary glycine intake and chronic diseases significantly different between young and old people but also between genders, especially for the association between dietary glycine intake and obesity. Strong correlation between reduced plasma glycine and increased VAT mass as markers of visceral obesity in older adults demonstrated (39). The findings of multiple prior studies indicate a negative correlation between glycine levels and the likelihood of developing type 2 diabetes in Germany and Finland, while no significant association has been identified among South Asians and British immigrants (40–42). However, it is also possible that the sample size was small and the results of the study were not stable enough.

Furthermore, the association of dietary glycine intake with hypertension and hyperlipidemia found in this study did not meet the expected results. Significance was found only overall and in the highest quartile group. In a metabolic syndrome model experiment, Hui-Wen Wu et al. found that glycine intervention significantly reduced hypertension and dyslipidemia, which showed that rats fed 8% fructose water and 8% fructose water +1% glycine water in groups showed an increasing trend in systolic blood pressure after 3 months of feeding in the former but a significant decrease in systolic blood pressure after 4–6 months of feeding in the latter. In the experimental model of Jing Liu et al., who showed that (43), in both normal feed and high-fat feed feeding, the injection of 20% glycine solution significantly inhibited the growth of body weight and fat. By looking at light microscopic sections of the liver, glycine had a protective effect on the livers of mice fed with high-fat chow and was able to attenuate high-fat diet-induced hepatic steatosis, and this effect was most pronounced in the livers of female mice, but the relationship between glycine and hyperlipidemia was not as significant in the population of this study. This may be due to some differences in the degree of response to glycine between humans and experimental animals, or it may be due to differences in dietary glycine intake versus glycine intervention alone, or the fact that this study was conducted on experimental animals in a metabolic syndrome model and did not take into account other factors such as hypertension due to other factors, such as spontaneous hypertension, salt-induced, and angiotensin-induced hypertensive rats.

There are several limitations to the current study, including the fact that it is cross-sectional and retrospective. However, it is also important to recognize that cross-sectional studies do not sufficiently confirm dietary factors’ causal relationship with disease and can sometimes even have the opposite effect (44). Therefore, long-term cohort studies are needed to confirm the relationships found in this study, and preliminary dietary interventions are also needed to adequately validate these associations. In addition, considering that China is a vast country with a large population and that the present study was limited to residents of poorer areas in northern China, further research on the relationship between dietary glycine intake and related chronic diseases on a larger scale is worth exploring. Despite many limitations, the present study also provided some valuable results. It has been found that higher intakes of dietary glycine reduced hypertension, overweight and obesity, and high cholesterol can help to provide dietary guidance to the residents of this locality and can guide the needy population to make certain dietary modifications to increase the dietary intake of glycine-rich protein foods and can be used to validate this finding.

## 5 Conclusion

The present investigation identified a relationship between elevated dietary glycine consumption and a diminished occurrence of hypertension, hyperlipidemia, and overweight/obesity, with a notable emphasis on overweight/obesity. Nevertheless, upon stratification by age or gender, the findings exhibited greater variability, and the association with the aforementioned conditions was attenuated. Additionally, the study’s sample size was relatively modest, necessitating further validation through population studies with larger cohorts. Subsequent dietary intervention recommendations are also warranted.

## Data availability statement

The original contributions presented in the study are included in the article/supplementary material, further inquiries can be directed to the corresponding author.

## Ethics statement

The studies involving humans were approved by Ethics Committee of Harbin Medical University. The studies were conducted in accordance with the local legislation and institutional requirements. The participants provided their written informed consent to participate in this study.

## Author contributions

YF: Data curation, Investigation, Writing – original draft, Writing – review & editing. XG: Methodology, Software, Writing – original draft, Writing – review & editing. ZM: Data curation, Investigation, Writing – review & editing. HW: Data curation, Investigation, Writing – review & editing. RF: Supervision, Writing – review & editing. ZZ: Conceptualization, Funding acquisition, Project administration, Supervision, Writing – review & editing.
